# Relationship between the microbiota in different sections of the gastrointestinal tract, and the body weight of broiler chickens

**DOI:** 10.1186/s40064-016-2604-8

**Published:** 2016-06-29

**Authors:** Geon Goo Han, Eun Bae Kim, Jinyoung Lee, Jun-Yeong Lee, Gwideuk Jin, Jongbin Park, Chul-Sung Huh, Ill-Kyong Kwon, Dong Yong Kil, Yun-Jaie Choi, Changsu Kong

**Affiliations:** Department of Agricultural Biotechnology, Seoul National University, Seoul, Republic of Korea; Department of Animal Life Science, Kangwon National University, Chuncheon, Gangwon-do Republic of Korea; Division of Applied Animal Science, Kangwon National University, Chuncheon, Gangwon-do Republic of Korea; Department of Animal Science and Technology, Konkuk University, Seoul, Republic of Korea; Department of Animal Life System, Kangwon National University, Chuncheon, Gangwon-do Republic of Korea; Institute of Green-Bio Science and Technology, Seoul National University, Pyeongchang, Gangwon-do Republic of Korea; Department of Animal Science and Technology, Chung-Ang University, Anseong, Gyeonggi-do Republic of Korea; Research Institute for Agriculture and Life Science, Seoul National University, Seoul, Republic of Korea

**Keywords:** Body weight, Broiler chickens, Gastrointestinal tract, Microbiota, Relationship

## Abstract

In the poultry industry, many efforts have been undertaken to further improve the growth performance of broilers and identification and modulation of body weight (BW)-related bacteria could be one of the strategies to improve productivity. However, studies regarding the relationship between microbiota and BW are scarce. The objective of the present study was to investigate the relationship between microbiota and BW in different sections of the gastrointestinal tract (GIT). A total of twenty 18-day-old birds were selected based on the BW, and samples were collected from the three different sections of the GIT, which included the crop, ileum and cecum. Bacterial genomic DNA was extracted from the samples, and the V4 region of 16S rRNA gene were amplified. Amplicons were sequenced on Illumina MiSeq, and microbial communities were analyzed by using QIIME. In principal coordinate analysis, bacterial communities were clustered into three groups, based on the sections of GIT. Several BW-related bacterial groups were identified from linear regression analysis. At the genus level, *Streptococcus* from the ileum as well as *Akkermansia* in both ileum and cecum, were negatively related to BW, whereas *Bifidobacterium* in the ileum and *Lactococcus* in the cecum showed a positive correlation. The results from the present study showed that particular bacterial communities in the GIT were related to BW, and the study has broadened the understanding of the intestinal microbial ecosystem in broiler chickens.

**Electronic supplementary material:**

The online version of this article (doi:10.1186/s40064-016-2604-8) contains supplementary material, which is available to authorized users.

## Background

In the broiler industry, productivity such as feed conversion ratio and growth rate has been improved for decades (Rubio et al. 2014); however, broiler chickens still seem to have a growth potential. Based on the consumer demand, strategies for increasing the market weight of broilers have been studied by many researchers, but an efficient approach has not yet been developed. Moreover, the use of antibiotics in animal feeds for growth promotion has been banned from January 2006 in the EU and from July 2011 in Korea, and this restriction has led to the development of efficient and safe antibiotic alternatives to enhance the growth performance of livestock animals, including broilers. In this current situation, development of efficient and economic strategies to improve the growth performance of broiler chickens are an important task in broiler industry, and understanding of the host-microbiota interaction is one of the possible strategies (Rinttila and Apajalahti 2013; Pedroso et al. 2012).

Development of high-throughput sequencing technology enabled culture-independent analysis of microbial communities, and several studies revealed that intestinal microbiota affects the host metabolism and physiology, such as metabolic homeostasis (Shin et al. 2011; Caricilli et al. 2011), angiogenesis (Reinhardt et al. 2012), obesity (Turnbaugh et al. 2009; Backhed et al. 2004), immune function (Ivanov and Littman 2011; Kuss et al. 2011; Ichinohe et al. 2011) and brain development (Diaz Heijtz et al. 2011). In the past decade, many studies were conducted to investigate the relationship between gut microbiota and body weight (BW). These studies revealed that in human and mice, certain phyla were positively correlated with BW (Delzenne and Cani 2011; Hildebrandt et al. 2009; Ley et al. 2006; Turnbaugh et al. 2009), while certain species were negatively correlated with BW (Everard et al. 2013; Santacruz et al. 2010; Everard et al. 2011; Santacruz et al. 2009). However, only a limited number of studies about relationship between intestinal microbiota and BW were conducted in livestock, especially in young animals. Identification and modulation of weight-related bacteria is one of the strategies to modulate BW, and it can potentially be a useful strategy to improve productivity in the broiler industry. Therefore, the objective of the present study was to investigate the relationship between the BW of broiler chickens and microbiota in different sections of the gastrointestinal tract (GIT).

## Results

### Microbial communities at different sections of GIT

**Fig. 1:**
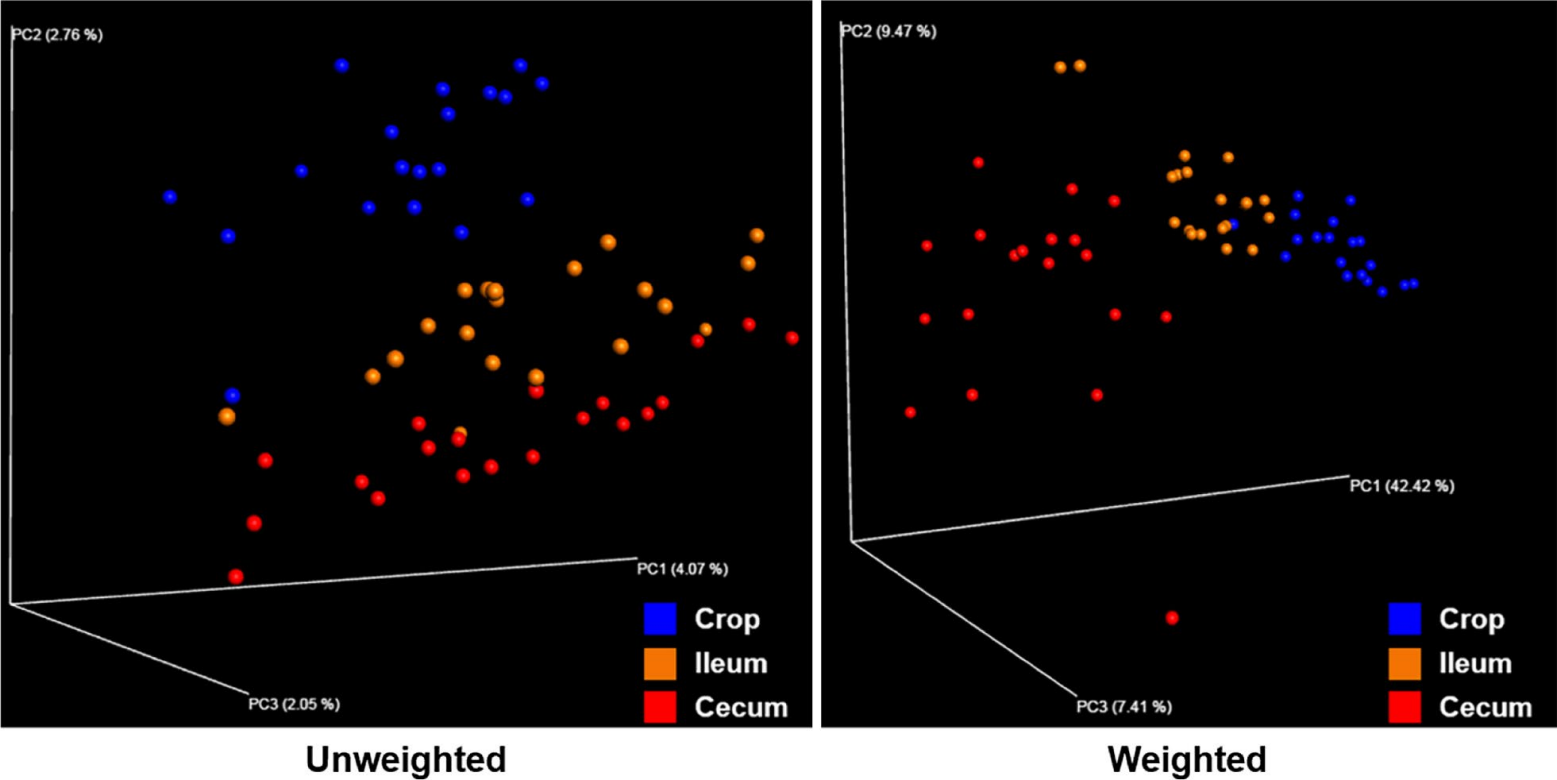
Principal coordinate analysis of unweighted and weighted UniFrac. Beta diversity patterns of crop samples (n = 19), ileal samples (n = 20) and cecal samples (n = 20) were explored using the principal coordinate analysis (PCoA). Subject color coding: *blue* crop samples; *yellow* ileal samples and *red* cecal samples

We compared the microbial communities in three sections of the GIT—crop, ileum and cecum. A total of 950,771 (mean = 16,115 ± 6460) 16S rRNA reads were generated, with an average of 18,085 (±5788) reads per crop sample, 15,294 (±6676) reads per ileal sample and 15,064 (±6733) reads per cecal sample. PCoA based on unweighted and weighted UniFrac distances of the 16S rRNA revealed that samples were clustered into three distinct groups (Fig. 1). In PCoA plot, microbial communities were separated by sections of the GIT, and ileal samples were placed between the crop and cecal samples.

To determine which bacterial taxa contributed to separate microbial communities, relative abundance of taxa in each section is shown in Tables 1 and 2, and it is represented as a heat map (Additional file 1: Fig. S1). At the phylum level, Cyanobacteria and Proteobacteria were significantly more abundant in the crop, and Firmicutes was significantly more abundant in the ileum than in other sections (Additional file 1: Fig. S2). At the genus level, *Bacillus* was significantly more abundant in the crop (Additional file 1: Fig. S3A) and *Prevotella* was significantly more abundant in the ileum than in other sections (Additional file 1: Fig. S3F). *Faecalibacterium*, *Ruminococcus* and *Akkermansia* were significantly more abundant, and *Lactobacillus* and *Streptococcus* were significantly less abundant, in the cecum than in other sections (Additional file 1: Fig. S3). Especially, genus *Bacteroides* showed an incremental increase from the cranial to the caudal section (crop < ileum < cecum).

**Table 1:** Relative abundance of phyla found in each section of the GIT

Phylum	Abundance (%)			SD^1^	*P* value
	Crop	Ileum	Cecum		
Cyanobacteria	12.89^a^	3.41^b^	1.34^b^	3.27	<0.001
Bacteroidetes	13.93^a^	22.15^b^	30.49^c^	5.82	<0.001
Proteobacteria	7.89^a^	4.26^b^	3.06^b^	2.18	<0.001
Tenericutes	0.85^a^	0.94^a^	1.63^b^	0.51	<0.001
Firmicutes	59.62^ab^	64.15^a^	58.37^b^	6.85	0.027
Euyarchaeota	0.08^a^	0.19^b^	0.16^ab^	0.12	0.033
Verrucomicrobia	0.09	0.06	0.22	0.21	0.057
Synergistetes	0.02	0.03	0.06	0.06	0.095
Lentisphaerae	0.02	0.04	0.03	0.02	0.118
Actinobacteria	1.18	0.95	1.05	0.35	0.133
Fibrobacteres	0.02	0.03	0.01	0.04	0.271
Spirochaetes	0.37	0.43	0.46	0.24	0.500
Fusobacteria	0.08	0.08	0.09	0.05	0.842
*n*	19	20	20		

One-way ANOVA with Tukey’s post hoc test was used. Within a row, different superscript letters indicate significant difference (*P* < 0.05)

^1^Pooled standard deviation

**Table 2:** Relative abundance of genera found in each section of the GIT

Genus	Abundance (%)			SD^1^	*P* value
	Crop	Ileum	Cecum		
*Bacillus*	4.34^a^	1.50^b^	1.37^b^	0.85	<0.001
*Bacteroides*	4.22^a^	7.54^b^	12.59^c^	2.66	<0.001
*Oscillospira*	1.00^a^	1.34^a^	2.38^b^	0.49	<0.001
*Faecalibacterium*	1.14^a^	1.47^a^	4.62^b^	1.30	<0.001
*Blautia*	0.22^a^	0.27^a^	0.44^b^	0.10	<0.001
*Dorea*	0.21^a^	0.30^a^	0.45^b^	0.12	<0.001
*Ruminococcus*	0.99^a^	1.24^a^	1.85^b^	0.43	<0.001
*Desulfovibrio*	0.09^a^	0.22^b^	0.13^a^	0.07	<0.001
*Lactobacillus*	28.62^a^	30.81^a^	18.12^b^	7.77	<0.001
*Bilophila*	0.03^a^	0.04^a^	0.10^b^	0.04	<0.001
*Coprococcus*	0.17^a^	0.23^a^	0.34^b^	0.11	<0.001
*Staphylococcus*	1.56^a^	1.18^a^	0.67^b^	0.60	<0.001
*Streptococcus*	1.18^a^	1.11^a^	0.75^b^	0.32	<0.001
*Clostridium*	0.96^a^	0.79^ab^	0.60^b^	0.27	<0.001
*Pediococcus*	0.07^a^	0.06^a^	0.04^b^	0.02	0.001
*Prevotella*	3.94^a^	5.66^b^	4.26^a^	1.55	0.003
*Akkermansia*	0.05^a^	0.04^a^	0.20^b^	0.20	0.033
*Methanobrevibacter*	0.08^a^	0.16^b^	0.15^ab^	0.11	0.045
*Selenomonas*	0.10^a^	0.09^ab^	0.06^b^	0.05	0.046
*Bifidobacterium*	0.84	0.61	0.77	0.33	0.077
*Enterococcus*	0.50	0.84	0.47	0.53	0.077
*Lactococcus*	0.09	0.05	0.03	0.16	0.489
*n*	19	20	20		

One-way ANOVA with Tukey’s post hoc test was used. Within a row, different superscript letters indicate significant difference (*P* < 0.05)

^1^Pooled standard deviation

### Relationship between alpha diversity and BW

**Fig. 2:**
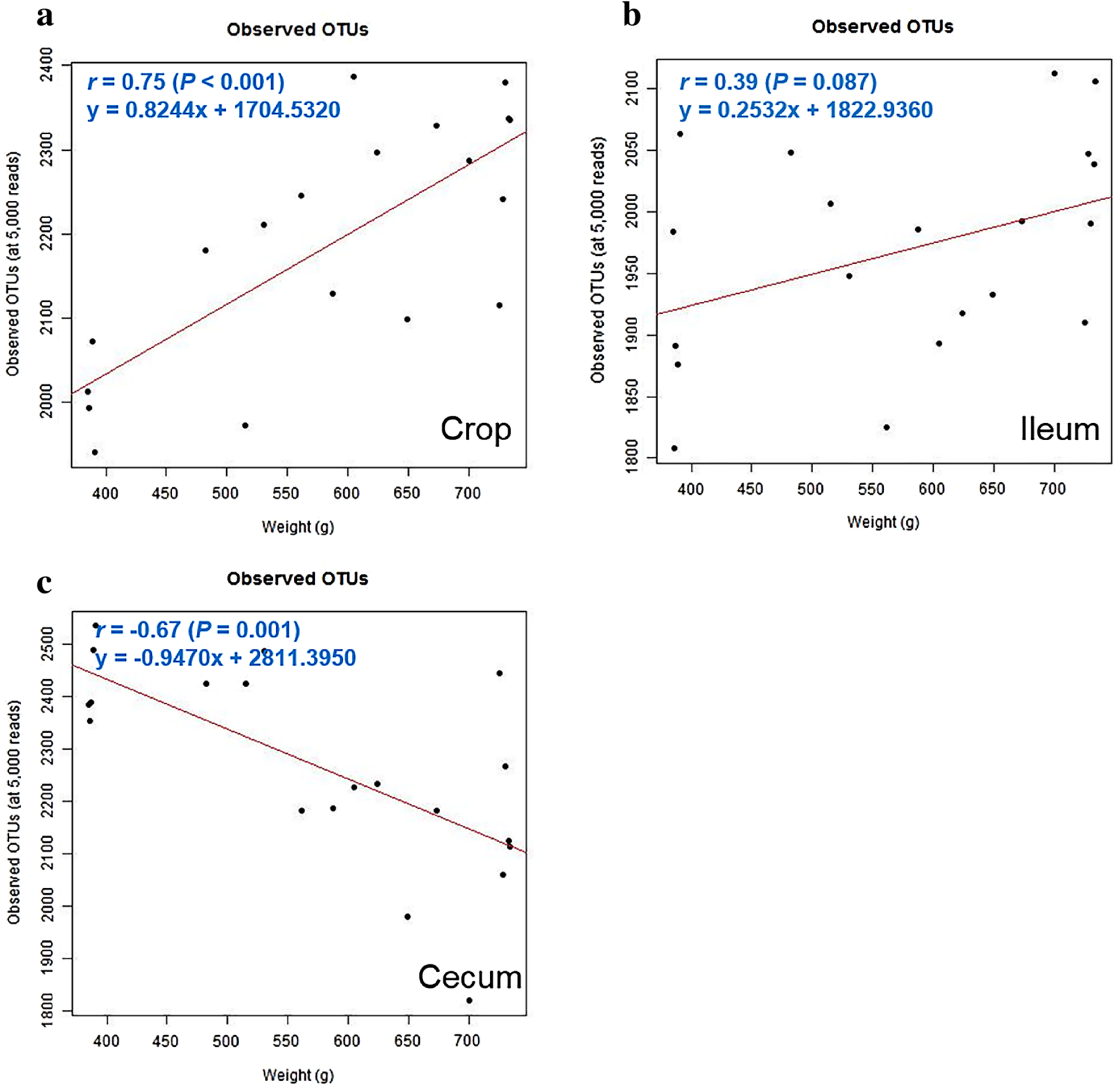
The relationship between BW and observed OTUs in chickens. The relationship was assessed by Pearson’s correlation coefficient (*r*) and *P* values from simple linear regression in the crop (**a**), ileum (**b**) and cecum (**c**)

In this study, the observed OTUs was used as a parameter of microbial diversity within the samples (alpha diversity). Relationship between observed OTUs and BW in each section was analyzed by linear regression analysis. Observed OTUs was positively correlated with BW in the crop (*r* = 0.75, *P* < 0.001) and the ileum (*r* = 0.39, *P* = 0.087) (Fig. 2a, b). Conversely, observed OTUs was negatively correlated with BW in the cecum (*r* = −0.67, *P* = 0.001) (Fig. 2c). These results suggest that microbial diversity positively correlates with BW in the crop and ileum, whereas it negatively correlates to BW in the cecum.

### BW related bacteria at each section of the GIT

A linear regression analysis was performed to determine which bacterial taxa were related to BW at each section of the GIT in the chicks. The overall significant results (*r* and *P* values) of analysis at the three sections are represented in Table 3. First, in the crop, weight related bacterial groups were explored (Additional file 1: Table S2). At the phylum level, Bacteroidetes (*r* = 0.66, *P* = 0.002) and Euryarchaeota (*r* = 0.52, *P* = 0.023) were positively related with BW, while Actinobacteria (*r* = −0.65, *P* = 0.003) was negatively related with BW. At the genus level, *Ruminococcus* (*r* = 0.72, *P* < 0.001) and *Faecalibacterium* (*r* = 0.65, *P* = 0.002) were positively related with BW, while *Bifidobacterium* (*r* = −0.64, *P* = 0.003) and *Lactobacillus* (*r* = −0.39, *P* = 0.099) were negatively related with BW.

**Table 3:** The relationship between BW and bacterial relative abundance in each section of the GIT

	Crop			Ileum			Cecum		
	Abundance (%)	*r*	*P*	Abundance (%)	*r*	*P*	Abundance (%)	*r*	*P*
Phylum									
Actinobacteria	1.18	−0.65	0.003	0.95	0.08	0.729	1.05	0.00	0.990
Bacteroidetes	13.93	0.66	0.002	22.15	0.30	0.192	30.49	<0.01	0.990
Euryarchaeota	0.08	0.52	0.023	0.19	0.52	0.018	0.16	0.14	0.558
Firmicutes	59.62	−0.21	0.392	64.15	−0.36	0.119	58.37	0.06	0.812
Proteobacteria	7.89	−0.17	0.482	4.26	0.10	0.672	3.06	−0.17	0.472
Spirochaetes	0.37	0.33	0.172	0.43	0.47	0.035	0.46	0.23	0.323
Verrucomicrobia	0.09	0.30	0.208	0.06	−0.10	0.690	0.22	−0.41	0.073
Genus									
*Akkermansia* ^a^	0.05	0.09	0.706	0.04	−0.51	0.023	0.08	−0.55	0.022
*Anaerovibrio*	0.35	0.20	0.413	0.44	−0.20	0.398	0.29	−0.81	<0.001
*Bacteroides*	4.22	0.54	0.016	7.54	0.05	0.834	12.59	−0.34	0.142
*Bifidobacterium*	0.84	−0.64	0.003	0.61	0.49	0.029	0.77	0.01	0.981
*Faecalibacterium*	1.14	0.65	0.003	1.47	0.23	0.335	4.62	0.32	0.164
*Lactobacillus*	28.62	−0.39	0.099	30.81	−0.32	0.172	18.12	0.02	0.924
*Lactococcus*	0.09	0.24	0.315	0.05	0.16	0.490	0.03	0.59	0.006
*Methanobrevibacter*	0.08	0.52	0.024	0.16	0.56	0.010	0.15	0.18	0.435
*Prevotella*	3.94	0.15	0.542	5.66	0.34	0.145	4.26	−0.59	0.006
*Ruminococcus*	0.99	0.72	<0.001	1.24	0.08	0.746	1.85	−0.35	0.132
*Streptococcus*	1.18	0.03	0.913	1.11	−0.81	<0.001	0.75	−0.06	0.787

*r* is Pearson’s correlation coefficient

^a^Three outliers were identified using Grubb’s test and removed from the cecum data

**Fig. 3:**
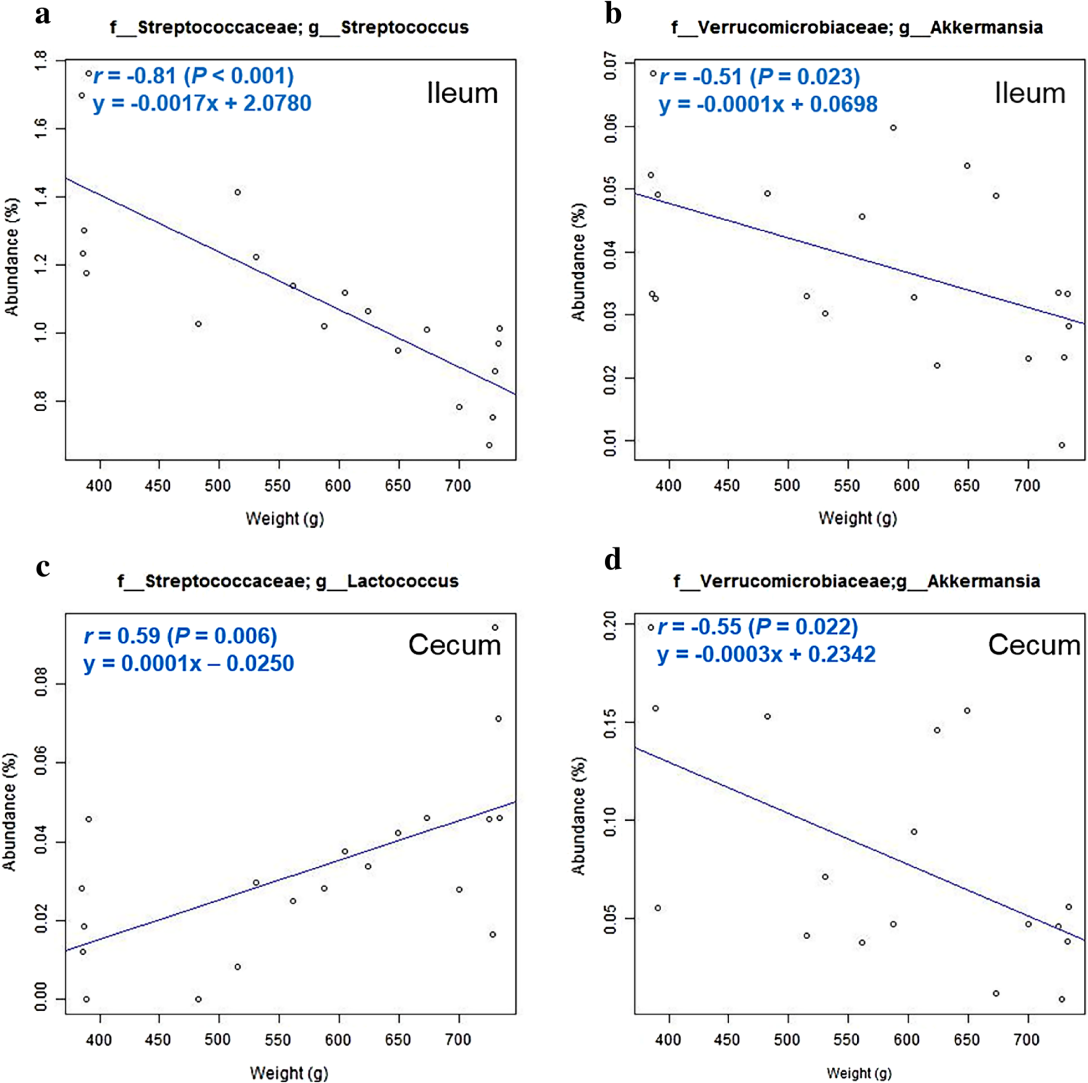
Weight related genera in the chicken GIT. *Streptococcus* (**a**) and *Akkermansia* (**b**) were significantly correlated with BW in the ileum. *Lactococcus* (**c**) and *Akkermansia* (**d**) were significantly correlated with BW in the cecum. **d** Three outliers were identified using Grubb’s test and removed from the dataset. The relationship between abundance of microbial taxa and BW was assessed by Pearson’s correlation coefficient (*r*) and *P* values from simple linear regression

Next, in the ileum, weight related bacterial groups were explored (Additional file 1: Table S3). At the phylum level, Euryarchaeota (*r* = 0.52, *P* = 0.018) and Spirochaetes (*r* = 0.47, *P* = 0.035) were positively related with BW. At the genus level, *Methanobrevibacter* (*r* = 0.56, *P* = 0.010) and *Bifidobacterium* (*r* = 0.49, *P* = 0.029) were positively related with BW, while *Akkermansia* (*r* = −0.51, *P* = 0.023) was negatively related with BW (Fig. 3b). Especially, *Streptococcus* (*r* = −0.81, *P* < 0.001) showed a strong negative correlation with BW (Fig. 3a).

Finally, weight related bacterial groups were explored in the cecum (Additional file 1: Table S4). At the phylum level, Lentisphaerae (*r* = −0.50, *P* = 0.023) and Verrucomicrobia (*r* = −0.41, *P* = 0.073) were negatively related with BW. At the genus level, *Lactococcus* (*r* = 0.59, *P* = 0.006) was positively related with BW (Fig. 3c), while *Anaerovibrio* (*r* = −0.81, *P* < 0.001)*, Prevotella* (*r* = −0.59, *P* = 0.006) and *Akkermansia* (*r* = −0.55, *P* = 0.022) were negatively related with BW (Fig. 3d).

## Discussion

In this study, we observed that microbial communities were clearly separated in different sections of the GIT in broiler chickens. Similar results were suggested by several previous researchers (Looft et al. 2014; Kamada et al. 2013; Sekelja et al. 2012; Videnska et al. 2013); for example, Looft et al. reported that ileum, cecum, mid-colon and feces have different microbial communities in swine at the phylum and genus level (Looft et al. 2014). Sekelja et al. also reported that a clear separation of microbial composition was seen between the upper gut (crop and gizzard), ileum and lower gut (cecum and colon) in broiler chickens (Sekelja et al. 2012). That these distinctions may be due to different nutrient requirements, is a critical factor for colonizing the commensal bacteria because each section has different nutrient factors (Deusch et al. 2015). The crop flora may be affected mainly by the microbial composition in the feed because the crop temporarily stores feed before digestion and is related to the breakdown of starch, whereas the ileal flora may be affected mainly by the nutrient composition of the ingested feed because ileum has a role involving the nutritional absorption of digested feed. The cecum is an anaerobic environment and plays an important role in recycling urea, absorption of water and digestion of plant structural carbohydrates, such as cellulose and hemicellulose. These varied features of each section of the GIT may result in the microbial community distinction.

Previous studies have revealed that the intestinal microbial diversity was negatively related with BW gain (Clarke et al. 2014; Turnbaugh et al. 2009). In our study, similar results were observed in the cecum, although the reverse was seen in the crop and ileum. Several studies suggested that reduced gut microbial diversity is correlated with several diseases such as inflammatory bowel disease and obesity-associated diabetes (Chang et al. 2008; Michail et al. 2012; Murri et al. 2013; Turnbaugh et al. 2009). While several other studies concluded that microbial diversity is not related with these diseases (Mejia-Leon et al. 2014; de Goffau et al. 2014; Walters et al. 2014), these results are controversial as of now, and thus, more studies are needed.

Several weight related bacterial groups in various region of the GIT were explored in this study. Especially, the genus *Streptococcus* showed a significant negative correlation with BW in the ileum. The genus *Streptococcus* can be divided into six groups, on the basis of 16S rRNA gene sequences—*S. anginosus* group, *S. bovis* group, *S. mitis* group, *S. mutans* group, *S. pyogenes* group and *S. salivarius* group (Kawamura et al. 1995). Many species of *Streptococcus* are known normal gut flora, however, some species are responsible for many diseases. For example, the *S. anginosus* group bacteria are associated with infections at multiple body sites, and abscess formation (Ruoff 1988; Belko et al. 2002; Bert et al. 1998). *S. mutans* group bacteria (Loesche 1986; Simon-Soro and Mira 2015) and *S. mitis* group bacteria (Mitchell 2011; Catto et al. 1987) are known pathogens of the buccal cavity. In humans, *S. acidominius* is a known pathogen that causes invasive diseases (Wu et al. 2014) such as pneumonia (Baker and Carlson 2008; Akaike et al. 1988), meningitis (Finkelstein et al. 2003) and brain abscess (Cone et al. 2007). Our data is consistent with the hypothesis that pathogenic *Streptococcus* might affect BW yet a more detailed analysis of the *Streptococcus* community is needed.

Recently many researchers revealed that *Akkermansia muciniphila*, the mucin degrading bacterium belonging to the genus *Akkermansia*, was negatively related to weight gain and obesity in mice and humans (Everard et al. 2013; Santacruz et al. 2010; Everard et al. 2011). Shin et al. also, reported that *A. muciniphila* has anti-diabetic potential against type 2 diabetes in high-fat diet fed mice (Shin et al. 2014). Similar results were obtained in our study. *Akkermansia* was inversely correlated with BW in the ileum and cecum, in spite of all birds having ingested the same feed. This suggests that the abundance of *Akkermansia* is more related to the BW, than the feed composition.

Many researchers reported that Firmicutes/Bacteroidetes (F/B) ratio increases when body mass index is increased, and F/B ratio is higher in the obese group than in the lean group (Delzenne and Cani 2011; Hildebrandt et al. 2009; Ley et al. 2006), although Clarke et al. suggested an opposing result (Clarke et al. 2014). In our study however, F/B ratio showed no significant relationship with BW in the ileum and cecum, although a negative relationship was observed in the crop (Additional file 1: Fig. S4). Moreover, Firmicutes and Bacteroidetes showed no significant correlation with BW in all parts of the GIT. More studies about the relationship between F/B ratio and BW are needed to explain these results.

## Conclusions

In this study, microbial communities were explored in various regions of the GIT, and several weight related bacterial groups were identified from linear regression analysis, such as the genus *Streptococcus* and genus *Akkermansia*. These results broaden our understanding of the microbial ecosystem and show that certain bacterial groups affect the BW of broiler chickens. Although more studies about the relationship between microbiota and BW are needed, these bacterial groups will be initial targets for improving the growth performance of broiler chickens.

## Methods

### Birds and sample preparation

A total of 545, day-old, male, Ross 308 broiler chicks (initial mean body weight = 38.5 g) were purchased from a local hatchery (Yangji hatchery, Pyeongtaek, Republic of Korea). The protocol for this experiment was reviewed and approved by the Institutional Animal Care and Use Committee at Chung-Ang University (IACUC #: 14-0005). Birds were provided with water and feed ad libitum from day 0 to day 17, housed in battery cages (76 cm × 78 cm × 45 cm = width × length × height for each cage). The cages were environmentally controlled, with continuous light. On day 0, birds were weighed and tagged, and fed the standard commercial starter diet until day 17 (Table 4). On day 17, a total of 20 birds were selected out of the 545 birds. For the selection, each of the five heaviest and lightest birds were selected, and from the remaining 535 birds, 10 birds were selected by BW spaced at equal intervals, with the BW ranging from 482 to 700 g (Additional file 1: Table S1). The total BW ranged from 385 to 734 g (mean ± SD = 575.9 ± 134.7 g) for the 20 selected chicks.

**Table 4:** Chemical composition of standard commercial starter diet

Item	Contents per kg
Metabolizable energy	3120 kcal
Crude protein	215 g
Fat (ether extract)	85.1 g
Crude fiber	32.0 g
Calcium	9.0 g
Phosphorous	6.7 g
Lysine	13.1 g
Sulfur-containing amino acids	10.3 g

On day 17, the 20 selected birds were euthanized by CO_2_ asphyxiation, and the digesta of the crop, ileum, and ceca were collected. The ileal digesta were collected from the distal two-third section of ileum from the Meckel’s diverticulum, to about 2 cm proximal to the ileocecal junction, through squeezing of the intestinal tract. All the samples collected from the birds were kept in a freezer at −20 °C for further analysis.

### DNA extraction and sequencing

DNA was extracted from the crop (n = 19, one sample was missing), ileal (n = 20) and cecal (n = 20) digesta (~250 mg) using NucleoSpin^®^Soil Kit (Macherey–Nagel, Düren, Germany), according to the manufacturer’s instructions, and stored at −20 °C. In this study, the V4 region of bacterial 16S rRNA gene was amplified from the total extracted DNA, because this region, which is commonly used in microbial community analysis, provides sufficient phylogenetic richness (Zhao et al. 2013; Caporaso et al. 2011). PCR amplification was performed using the Takara Ex-taq polymerase (Takara Bio, Shiga, Japan) and universal primers (forward: 5′-GGACTACHVGGGTWTCTAAT-3′, reverse: 5′-GTGCCAGCMGCCGCGGTAA-3′). The amplification program consisted of one cycle of 94 °C for 3 min; 40 cycles of 94 °C for 45 s, 55 °C for 1 min, and 72 °C for 1.5 min; and finally one cycle of 72 °C for 10 min. Amplicons were separated by gel electrophoresis and purified using QIAquick Gel Extraction Kit (Qiagen, CA, USA).

For Illumina sequencing, DNA library was constructed using NEBNext Ultra DNA Library Prep Kit for Illumina (New England BioLabs, MA, USA), with some modifications of the manufacturer’s instructions. The size selection of adaptor-ligated DNA and cleanup of PCR amplification steps were replaced with PCR purification using a QIAquick PCR Purification Kit (Qiagen, CA, USA). Adaptor ligation and index primer addition were performed using NEBNext Multiplex Oligos for Illumina (New England BioLabs, MA, USA). DNA library construction was confirmed by agarose gel electrophoresis, and amplicons were purified using QIAquick Gel Extraction Kit. They were then sequenced on Illumina MiSeq platform (NICEM, SNU, Seoul, Republic of Korea). The 16S rRNA gene sequences obtained from MiSeq were deposited into NCBI’s Sequence Read Archive (SRA) database with accession number SRP065823.

### Microbial community analysis

Microbial community was analyzed by using Quantitative Insights Into Microbial Ecology (QIIME) version 1.9.0 (http://qiime.org). Raw sequence reads were quality filtered and demultiplexed. The sequence reads were clustered into operational taxonomic units (OTUs) at 97 % similarity using the Greengene database. Microbial diversity was assessed within samples (alpha diversity) or between samples (beta diversity) using QIIME. Alpha diversity (observed OTUs) was calculated through rarefaction with ten iterations. Beta diversity was calculated on the sequence reads based on weighted and unweighted UniFrac distance matrices. Principal coordinate analysis (PCoA) was performed based on UniFrac distances and visualized with EMPeror Software (Vazquez-Baeza et al. 2013).

### Statistical analysis

Statistical analysis was performed with R statistical package (version 3.0.3) (R Foundation for Statistical Computing, Vienna, Austria). Abundance of microbial taxa was expressed as percentage of total 16S rRNA gene sequences. One-way analysis of variance (ANOVA) and post hoc Tukey’s HSD test for multiple mean comparisons were used to find significant differences in microbial taxa among each section of the GIT. Significance was assumed at *P* < 0.05. The relationship between abundance of microbial taxa and BW was assessed by Pearson’s correlation coefficient (*r*) and *P* values from simple linear regression.

## Additional file

10.1186/s40064-016-2604-8 Heat map of the relative abundance of taxa in each sample at the phylum level (A) and at the genus level (B). A range of colors, from *green* to *red*, indicates the prevalence of each taxon. Taxa are sorted in ascending order by P values from one-way ANOVA test. **Figure S2.** Relative abundance of phyla found in each section of the GI tract. (A) Cyanobacteria, (B) Bacteroidetes, (C) Proteobacteria, (D) Firmicutes. Different superscript letters indicate statistical significance (P < 0.05). One-way ANOVA with Tukey’s post hoc test was used to find significant differences of relative abundance. **Figure S3.** Relative abundance of genera found in each section of the GI tract. (A) Bacillus, (B) Bacteroides, (C) Faecalibacterium, (D) Ruminococcus, (E) Lactobacillus, (F) Prevotella, (G) Streptococcus, (H) Akkermansia. Different superscript letters indicate statistical significance (P < 0.05). One-way ANOVA with Tukey’s post hoc test was used to find significant differences of relative abundance. **Figure S4.** The relationship between body weight and Firmicutes/Bacteroidetes (F/B) ratio. (A) Crop, (B) Ileum, (C) Cecum. The relationship between abundance of microbial taxa and BW was assessed by Pearson’s correlation coefficient (r) and P values from simple linear regression. **Table S1.** Sample information in this study. **Table S2.** The relationship between body weight and bacterial abundance in crop. **Table S3.** The relationship between body weight and bacterial abundance in ileum. **Table S4.** The relationship between body weight and bacterial abundance in cecum.
