# Supplementary material for: Relationship between the microbiota in different sections of the gastrointestinal tract, and the body weight of broiler chickens
Source: Springerplus. 2016 Jun 29;5(1):911. doi: 10.1186/s40064-016-2604-8 (PMC4927549; doi:10.1186/s40064-016-2604-8)
Supplement: Supplementary file 1 — 10.1186/s40064-016-2604-8 Heat map of the relative abundance of taxa in each sample at the phylum level (A) and at the genus level (B). A range of colors, from green to red, indicates the prevalence of each taxon. Taxa are sorted in ascending order by P values from one-way ANOVA test. Figure S2. Relative abundance of phyla found in each section of the GI tract. (A) Cyanobacteria, (B) Bacteroidetes, (C) Proteobacteria, (D) Firmicutes. Different superscript letters indicate statistical significance (P < 0.05). One-way ANOVA with Tukey’s post hoc test was used to find significant differences of relative abundance. Figure S3. Relative abundance of genera found in each section of the GI tract. (A) Bacillus, (B) Bacteroides, (C) Faecalibacterium, (D) Ruminococcus, (E) Lactobacillus, (F) Prevotella, (G) Streptococcus, (H) Akkermansia. Different superscript letters indicate statistical significance (P < 0.05). One-way ANOVA with Tukey’s post hoc test was used to find significant differences of relative abundance. Figure S4. The relationship between body weight and Firmicutes/Bacteroidetes (F/B) ratio. (A) Crop, (B) Ileum, (C) Cecum. The relationship between abundance of microbial taxa and BW was assessed by Pearson’s correlation coefficient (r) and P values from simple linear regression. Table S1. Sample information in this study. Table S2. The relationship between body weight and bacterial abundance in crop. Table S3. The relationship between body weight and bacterial abundance in ileum. Table S4. The relationship between body weight and bacterial abundance in cecum. [file 40064_2016_2604_MOESM1_ESM.docx]

**Supplementary information**

**Supplementary figure legends**

**Figure S1. Heat map of the relative abundance of taxa in each sample at the phylum level (A) and at the genus level (B).** A range of colors, from green to red, indicates the prevalence of each taxon. Taxa are sorted in ascending order by *P* values from one-way ANOVA test

**Figure S2. Relative abundance of phyla found in each section of the GI tract.** (A) Cyanobacteria, (B) Bacteroidetes, (C) Proteobacteria, (D) Firmicutes. Different superscript letters indicate statistical significance (*P* < 0.05). One-way ANOVA with Tukey’s post hoc test was used to find significant differences of relative abundance

**Figure S3. Relative abundance of genera found in each section of the GI tract.** (A) *Bacillus,* (B) *Bacteroides,* (C) *Faecalibacterium,* (D) *Ruminococcus,* (E) *Lactobacillus,* (F) *Prevotella,* (G) *Streptococcus,* (H) *Akkermansia*. Different superscript letters indicate statistical significance (*P* < 0.05). One-way ANOVA with Tukey’s post hoc test was used to find significant differences of relative abundance

**Figure S4. The relationship between body weight and Firmicutes/Bacteroidetes (F/B) ratio.** (A) Crop, (B) Ileum, (C) Cecum. The relationship between abundance of microbial taxa and BW was assessed by Pearson’s correlation coefficient (*r*) and *P* values from simple linear regression


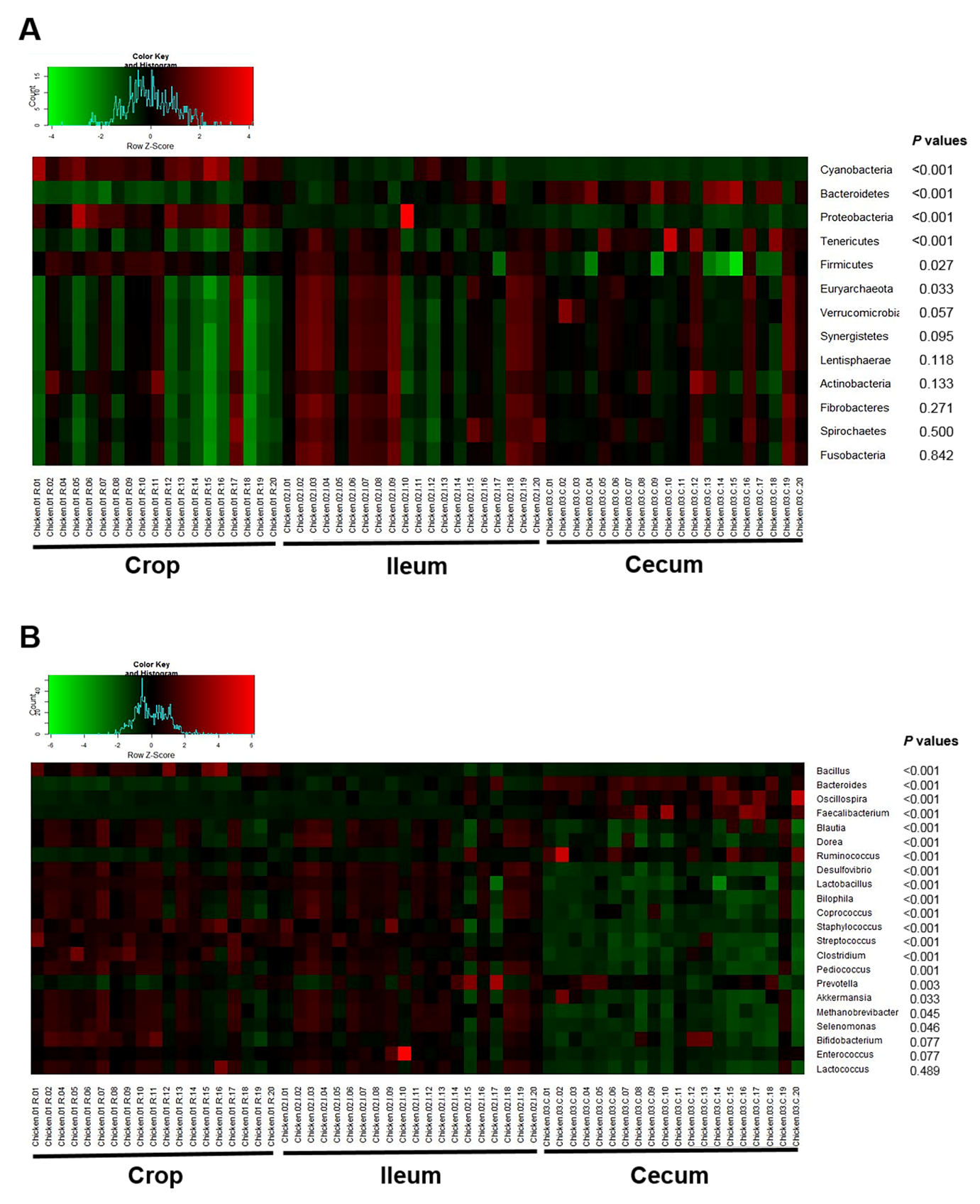


Figure S1


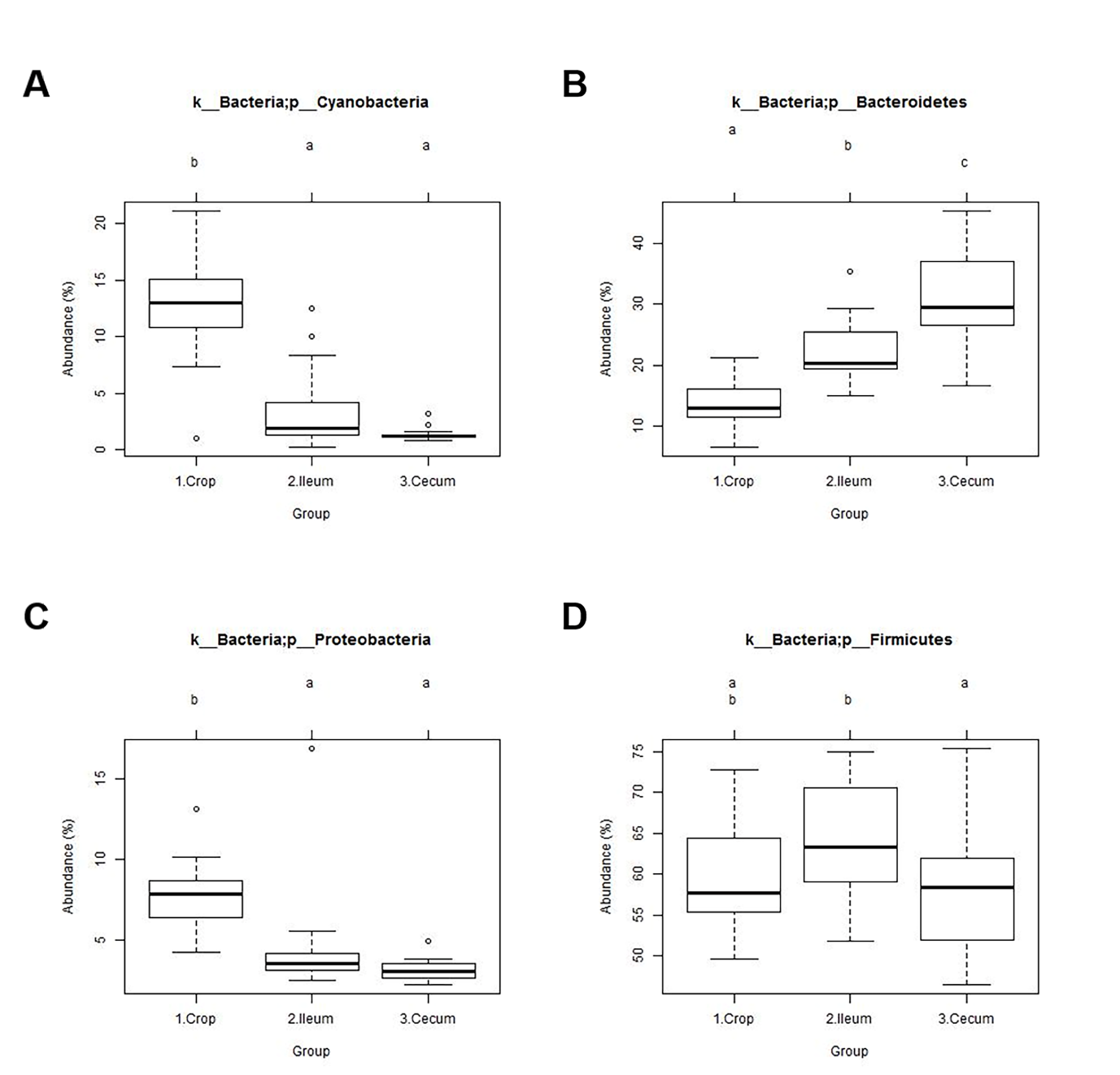


Figure S2


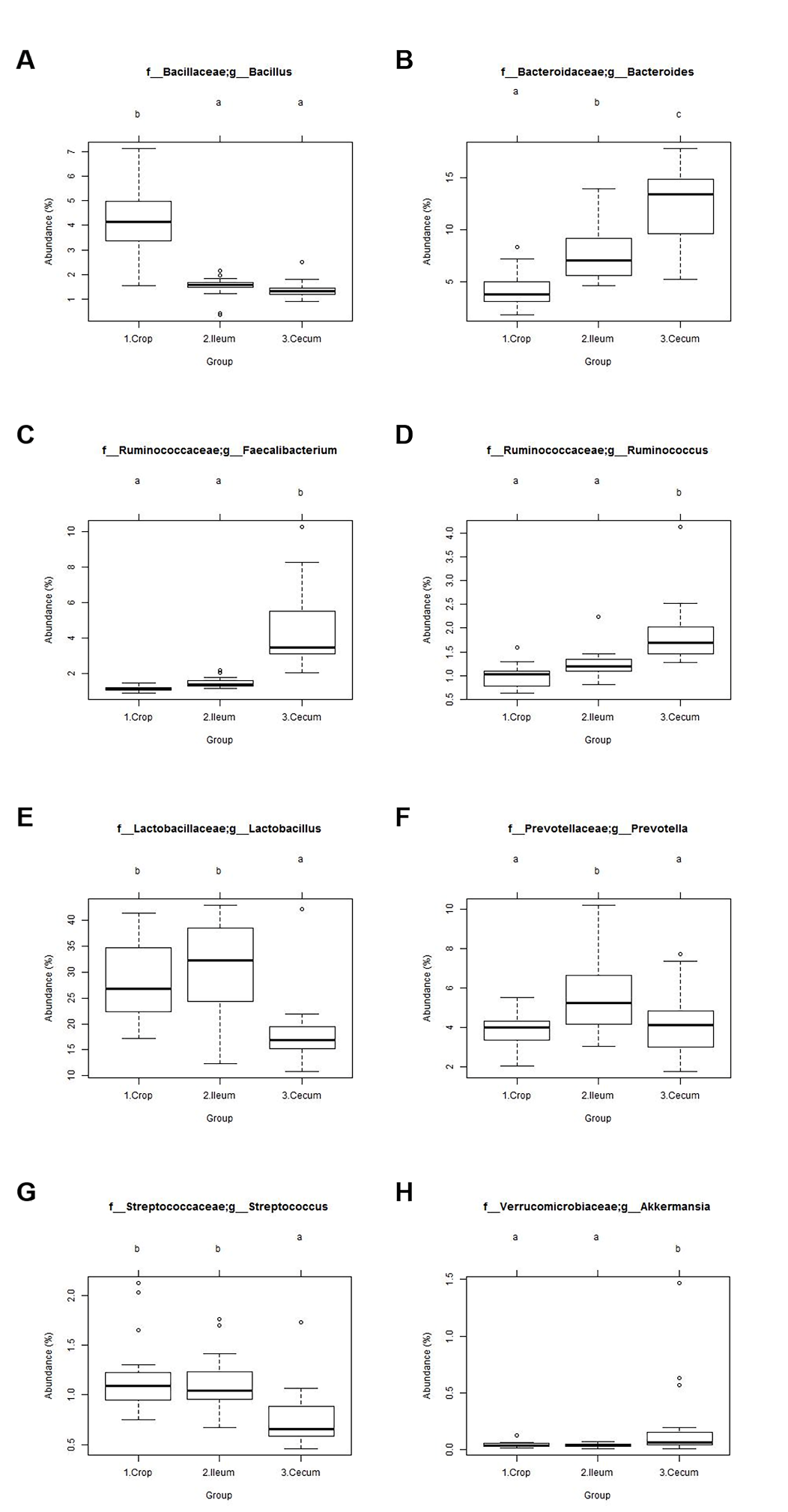


Figure S3


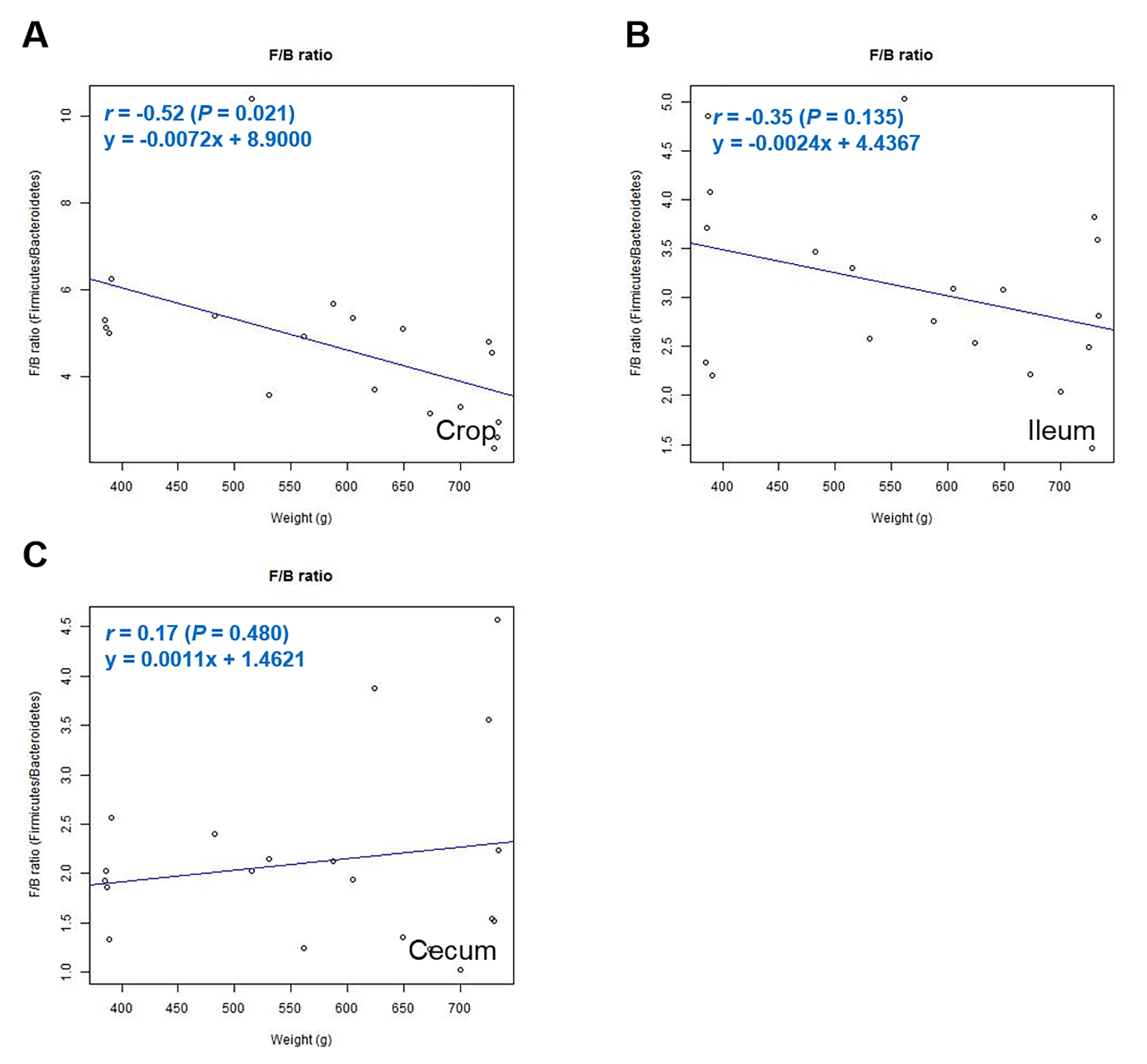


Figure S4

Supplementary tables

Table S1 Sample information in this study

| **Sample** | **Sample key** | | | **Body weight, g** | **Age, day** |
| --- | --- | --- | --- | --- | --- |
|  | **Crop** | **Ileum** | **Cecum** |  |  |
| 1 | R-1 | I-1 | C-1 | 385 | 18 |
| 2 | R-2 | I-2 | C-2 | 386 | 18 |
| 3 | - | I-3 | C-3 | 387 | 18 |
| 4 | R-4 | I-4 | C-4 | 389 | 18 |
| 5 | R-5 | I-5 | C-5 | 391 | 18 |
| 6 | R-6 | I-6 | C-6 | 482 | 18 |
| 7 | R-7 | I-7 | C-7 | 515 | 18 |
| 8 | R-8 | I-8 | C-8 | 531 | 18 |
| 9 | R-9 | I-9 | C-9 | 562 | 18 |
| 10 | R-10 | I-10 | C-10 | 588 | 18 |
| 11 | R-11 | I-11 | C-11 | 605 | 18 |
| 12 | R-12 | I-12 | C-12 | 624 | 18 |
| 13 | R-13 | I-13 | C-13 | 649 | 18 |
| 14 | R-14 | I-14 | C-14 | 673 | 18 |
| 15 | R-15 | I-15 | C-15 | 700 | 18 |
| 16 | R-16 | I-16 | C-16 | 726 | 18 |
| 17 | R-17 | I-17 | C-17 | 728 | 18 |
| 18 | R-18 | I-18 | C-18 | 730 | 18 |
| 19 | R-19 | I-19 | C-19 | 733 | 18 |
| 20 | R-20 | I-20 | C-20 | 734 | 18 |

Table S2 The relationship between body weight and bacterial abundance in crop

| **Bacteria** | ***r*^1^** | ***P***^2^ |
| --- | --- | --- |
| ***Phylum*** |  |  |
| Bacteroidetes | 0.66 | 0.002 |
| Actinobacteria | -0.65 | 0.003 |
| Euryarchaeota | 0.52 | 0.023 |
| Elusimicrobia | 0.44 | 0.061 |
| Synergistetes | 0.41 | 0.083 |
| Verrucomicrobia | 0.30 | 0.208 |
| Firmicutes | -0.21 | 0.392 |
| Proteobacteria | -0.17 | 0.482 |
|  |  |  |
| ***Genus*** |  |  |
| *Ruminococcus* | 0.72 | 0.001 |
| *Oscillospira* | 0.70 | 0.001 |
| *Propionibacterium* | -0.66 | 0.002 |
| *Parabacteroides* | 0.65 | 0.002 |
| *Faecalibacterium* | 0.65 | 0.003 |
| *Bifidobacterium* | -0.64 | 0.003 |
| *Catenibacterium* | -0.62 | 0.005 |
| *Anaerobacillus* | 0.56 | 0.013 |
| *Anaerotruncus* | 0.55 | 0.015 |
| *Bilophila* | 0.55 | 0.015 |
| *Aerococcus* | -0.55 | 0.016 |
| *Bacteroides* | 0.54 | 0.016 |
| *Brachybacterium* | -0.54 | 0.017 |
| *Odoribacter* | 0.53 | 0.019 |
| *Methanobrevibacter* | 0.52 | 0.024 |
| *Butyricimonas* | 0.50 | 0.028 |
| *Lactobacillus* | -0.39 | 0.099 |

^1^ *r* is Pearson’s correlation coefficient

^2^ Data were sorted in ascending order by *P* values from linear regression analysis

Table S3 The relationship between body weight and bacterial abundance in ileum

| **Bacteria** | ***r*^1^** | ***P***^2^ |
| --- | --- | --- |
| ***Phylum*** |  |  |
| Euryarchaeota | 0.52 | 0.018 |
| Spirochaetes | 0.47 | 0.035 |
| Firmicutes | -0.36 | 0.119 |
| Bacteroidetes | 0.30 | 0.192 |
| Proteobacteria | 0.10 | 0.672 |
| Verrucomicrobia | -0.10 | 0.690 |
|  |  |  |
| ***Genus*** |  |  |
| *Streptococcus* | -0.81 | < 0.001 |
| *Phascolarctobacterium* | 0.65 | 0.002 |
| *Marinibacillus* | -0.63 | 0.003 |
| *Methanobrevibacter* | 0.56 | 0.010 |
| *Pseudoramibacter_Eubacterium* | 0.54 | 0.013 |
| *RFN20* | 0.53 | 0.016 |
| *Akkermansia* | -0.51 | 0.023 |
| *Granulicatella* | -0.50 | 0.023 |
| *Treponema* | 0.50 | 0.025 |
| *Bifidobacterium* | 0.49 | 0.029 |
| *Trichococcus* | -0.48 | 0.034 |
| *Coprobacillus* | -0.47 | 0.035 |
| *Atopobium* | -0.47 | 0.036 |
| *Gracilibacter* | -0.47 | 0.036 |
| *Proteus* | -0.45 | 0.049 |

^1^ *r* is Pearson’s correlation coefficient

^2^ Data were sorted in ascending order by *P* values from linear regression analysis

Table S4 The relationship between body weight and bacterial abundance in cecum

| **Bacteria** | ***r*^1^** | ***P***^2^ |
| --- | --- | --- |
| ***Phylum*** |  |  |
| Elusimicrobia | -0.59 | 0.006 |
| Lentisphaerae | -0.50 | 0.023 |
| Verrucomicrobia | -0.41 | 0.073 |
| Spirochaetes | 0.23 | 0.323 |
| Proteobacteria | -0.17 | 0.472 |
| Euryarchaeota | 0.14 | 0.558 |
| Firmicutes | 0.06 | 0.812 |
| Bacteroidetes | 0.00 | 0.990 |
|  |  |  |
| ***Genus*** |  |  |
| *Anaerovibrio* | -0.81 | < 0.001 |
| *Pasteurella* | -0.73 | < 0.001 |
| *CF231* | -0.66 | 0.002 |
| *Lactococcus* | 0.59 | 0.006 |
| *Prevotella* | -0.59 | 0.006 |
| *Megamonas* | -0.58 | 0.008 |
| *Dialister* | -0.57 | 0.008 |
| *Shuttleworthia* | -0.52 | 0.019 |
| *Dehalobacterium* | -0.52 | 0.020 |
| *Butyricimonas* | -0.52 | 0.020 |
| *Akkermansia* | -0.41 | 0.074 |
| *Bacteroides* | -0.34 | 0.142 |
| *Faecalibacterium* | 0.32 | 0.164 |
| *Enterococcus* | 0.31 | 0.189 |
| *Methanobrevibacter* | 0.18 | 0.435 |
| *Clostridium* | -0.14 | 0.566 |
| *Streptococcus* | -0.06 | 0.787 |
| *Lactobacillus* | 0.02 | 0.924 |
| *Bifidobacterium* | 0.01 | 0.981 |

^1^ *r* is Pearson’s correlation coefficient

^2^ Data were sorted in ascending order by *P* values from linear regression analysis
